# Patient-reported diagnostic intervals to colorectal cancer diagnosis in the Midland region of New Zealand: a prospective cohort study

**DOI:** 10.1093/fampra/cmab155

**Published:** 2021-12-06

**Authors:** Tania Blackmore, Lynne Chepulis, Keenan Rawiri, Jacquie Kidd, Tim Stokes, Melissa Firth, Mark Elwood, David Weller, Jon Emery, Ross Lawrenson

**Affiliations:** Medical Research Centre, University of Waikato, Hamilton, New Zealand; Medical Research Centre, University of Waikato, Hamilton, New Zealand; Medical Research Centre, University of Waikato, Hamilton, New Zealand; Auckland University of Technology, Auckland, New Zealand; Department of General Practice and Rural Health, University of Otago, Dunedin, New Zealand; Medical Research Centre, University of Waikato, Hamilton, New Zealand; School of Population Health, University of Auckland, Auckland, New Zealand; Centre for Population Health Studies, The University of Edinburgh, Edinburgh, Scotland, UK; Medicine, Dentistry and Health Sciences, The University of Melbourne, Melbourne, VIC, Australia; Medical Research Centre, University of Waikato, Hamilton, New Zealand

**Keywords:** colorectal cancer, general practice, delayed diagnosis, bowel, New Zealand, questionnaire

## Abstract

**Background and objectives:**

New Zealand (NZ) has high rates of colorectal cancer (CRC) but low rates of early detection. The majority of CRC is diagnosed through general practice, where lengthy diagnostic intervals are common. We investigated factors contributing to diagnostic delay in a cohort of patients newly diagnosed with CRC.

**Methods:**

Patients were recruited from the Midland region and interviewed about their diagnostic experience using a questionnaire based on a modified Model of Pathways to Treatment framework and SYMPTOM questionnaire. Descriptive statistics were used to describe the population characteristics. Chi-square analysis and logistic regression were used to analyse factors influencing diagnostic intervals.

**Results:**

Data from 176 patients were analysed, of which 65 (36.9%) experienced a general practitioner (GP) diagnostic interval of >120 days and 96 (54.5%) experienced a total diagnostic interval (TDI) > 120 days. Patients reporting rectal bleeding were less likely to experience a long TDI (odds ratio [OR] 0.34, 95% confidence interval [CI]: 0.14–0.78) and appraisal/help-seeking interval (OR, 0.19, 95% CI: 0.06–0.59). Patients <60 were more likely to report a longer appraisal/help-seeking interval (OR, 3.32, 95% CI: 1.17–9.46). Female (OR, 2.19, 95% CI: 1.08–4.44) and Māori patients (OR, 3.18, 95% CI: 1.04–9.78) were more likely to experience a long GP diagnostic interval.

**Conclusion:**

NZ patients with CRC can experience long diagnostic intervals, attributed to patient and health system factors. Young patients, Māori, females, and patients experiencing change of bowel habit may be at particular risk. We need to increase symptom awareness of CRC for patients and GPs. Concentrated efforts are needed to ensure equity for Māori in access to screening, diagnostics, and treatment.

**Key messages**

- New Zealand patients newly diagnosed with colorectal cancer (CRC) can experience long diagnostic intervals.
- Young patients, Māori, and female patients experience longer intervals.
- Changes in bowel habit are also associated with longer time to diagnosis.
- We need to increase CRC symptom awareness for patients and general practice.

## Background

Colorectal cancer (CRC) is the second most common cancer in New Zealand (NZ),^1^ with over 3,000 newly registered cases and approximately 1,200 deaths in 2018.^2^ The GLOBOCAN age-standardized estimated incidence rate places Australia and NZ as having the highest rates of CRC in the world, with an incidence of 33.2 per 100,000 and mortality of 9.5 per 100,000.^3^ However, NZ has a low rate of early stage CRC diagnosis with fewer than 12% of patients diagnosed at stage I.^4^ This is attributed, in part, to the absence of a nationwide screening programme which was implemented for 60–74 year olds by each of the 20 District Health Boards (DHBs) in NZ. A recent systematic review^5^ also highlighted a paucity of information regarding outcomes for Māori—the indigenous peoples of NZ—despite known inequity and survival disparities.^6^ In the absence of screening, the majority of NZ CRC cases are diagnosed through symptomatic presentation to general practice. Delays to CRC diagnosis are common in general practice, with lengthy diagnostic intervals constituting 27% of complaints to the Health and Disability Commissioner (HDC) (2004–2013).^7^ Factors associated with long times to diagnosis are multifactorial^8^ and involve symptom characteristics, patient and health system factors. These factors can be considered according to the Model of Pathways to Treatment (MPT),^9^ a theoretical framework that can be applied to understand factors influencing patient pathways to diagnosis. The MPT outlines 4 phases of potential delay from first symptom recognition to start of treatment (the appraisal, help seeking, diagnostic, and pre-treatment intervals) and has been used to understand patient pathways in previous cancer research.^10–13^

Importantly, CRC is more difficult to diagnose in terms of its presenting symptoms than other cancers.^14,15^ The appraisal interval, where patients recognize that symptoms need medical investigation, has high potential for delay.^8^ Common symptoms include rectal bleeding, abdominal pain, and a change of bowel habit (COBH) (either sudden onset diarrhoea or constipation),^16^ but these symptoms also occur widely in the general population,^17^ and are often a result of more benign conditions such as haemorrhoids or irritable bowel syndrome (IBS). Difficulty in recognizing the potential seriousness of symptoms contributes to long appraisal intervals, especially if symptoms are intermittent and have been previously experienced or considered “normal.” Subsequently, patients often postpone help-seeking, choose to self-manage, or wait for symptom resolution, only consulting a general practitioner (GP) when symptoms have worsened,^18^ or as with bowel symptoms, might never consult their GP.^19^ After symptom appraisal, patients move to the help-seeking phase of the MPT, where they must overcome a number of barriers to consulting their GP, such as fear of tests,^8^ worry about what investigations might find,^16^ symptom embarrassment,^20^ or not wanting to bother the doctor.^21^ The quality of the patient–GP relationship^22^ and poor continuity of care also impede GP consultations.^20^ Young patients might postpone help-seeking if they perceive that they are too young for symptoms to be cancer related.^8^

GPs manage the patient transition to the diagnostic phase of the MPT, where symptoms lead to further investigations, specialist referral, and eventual diagnosis. GPs face a difficult task differentiating presenting symptoms that may be due to cancer from benign conditions, and must interpret symptoms while considering patient medical history and comorbid conditions. Comorbidity especially complicates accurate diagnosis,^23^ particularly if conditions are gastrointestinal (GI) in nature (e.g., diverticulitis, IBS). Furthermore, CRC is not common in general practice, with GPs typically diagnosing one patient per year.^24^ With a CRC diagnosis being rare, more common diagnoses are often considered first, especially in the light of existing GI issues or other comorbidity,^25^ which may lead to further delay and multiple GP consultations.^14^ GP–patient communication is vital. Long intervals can be due to GPs reassuring patients not to worry,^26^ advising to wait and self-monitor symptoms,^9^ or not take symptoms seriously.^27^ Furthermore, even if a GP recognizes further investigation *is* warranted, a 2016 survey showed that compared to Australia, Canada, the United Kingdom, Denmark, Norway, and Sweden, NZ GPs have less and slower access to investigative tests such as colonoscopy, CT, MRI, and flexible sigmoidoscopy.^28^

With low rates of early stage CRC diagnosis in NZ,^3^ we aimed to investigate factors associated with prolonged diagnostic intervals in a cohort of patients newly diagnosed with CRC from the Midland region.

## Methods

### Patient recruitment

NZ is divided into 20 DHBs and 4 regional cancer networks: the Northern, Midland, Central, and Southern. Patients were recruited from 3 of the 4 DHBs from the Midland Cancer region, including Waikato (population: 400,000+), Tairawhiti (population: 40,000+), and Lakes (population: 100,000+) District Health Boards (DHBs). Patients were initially recruited through referral from a cancer nurse specialist (CNS) employed at each DHB and then contacted via telephone for interview to complete a structured questionnaire. Additional recruitment occurred via mail out of study information using DHB clinic lists, a poster placed at Waikato hospital and private consulting rooms, and Bowel Cancer NZs social media page. No interviews to collect questionnaire data occurred without patient consent. Patients were eligible for recruitment if they had been diagnosed within 12 months (study period from 2016 to 2019) and had not been diagnosed through regional screening. Interviews were held from April 2018 to March 2020 and were usually carried out via telephone (or CNS at Lakes DHB). Interviews were occasionally conducted at Waikato DHB or at the patient’s home by prior arrangement. Ethical approval for this study was granted by the New Zealand Health and Disability Ethics Committee (Ref: 17/NTB/156).

### Data collection

Data were collected via interview to deliver a structured 52-item questionnaire based on the MPT^9^ and a modified SYMPTOM questionnaire.^25^ Questions were a mixture of reporting of dates, yes/no tick boxes, and Likert style ratings. Six questions were styled as open. The questionnaire was uploaded to a web-based survey tool (CrowdSignal) for delivery via iPad. During the interview, patients were invited to speak about their diagnostic experience, focussing on symptoms and the timeline from symptom onset to when a health care professional (usually a GP) was consulted to confirm the diagnosis. Additional questions captured the patient experience with their primary health care provider. Patient-reported comorbidities were recorded: i.e., asthma, chronic obstructive pulmonary disease, other lung issues, heart disease, anxiety or depression, inflammatory bowel disease, IBS, peptic ulcer, previous cancer, diabetes, and arthritis. Comorbidities were combined and recorded as 0 or 1+ for analysis. Diagnostic pathway included GP referral, hospital emergency department (ED), incidental (as a result of GP or hospital testing/procedure(s) for other conditions, e.g., routine blood tests, preparation for a non-related CRC surgery, heart investigations), and other (self-referral to specialists, being monitored for CRC, or other conditions).

Dates of first symptom presentation and first GP presentation were patient reported. Exact patient-reported dates were used, but if inexact dates were given an estimated date was used (e.g. “May 2018” was recorded as the midpoint of that month (e.g. 15 May 2018)). For Waikato patients who were uncertain of their diagnosis date, clinical records at Waikato DHB were accessed, and date of colonoscopy was estimated as the date of diagnosis.

### Delay intervals

**Figure 1.**
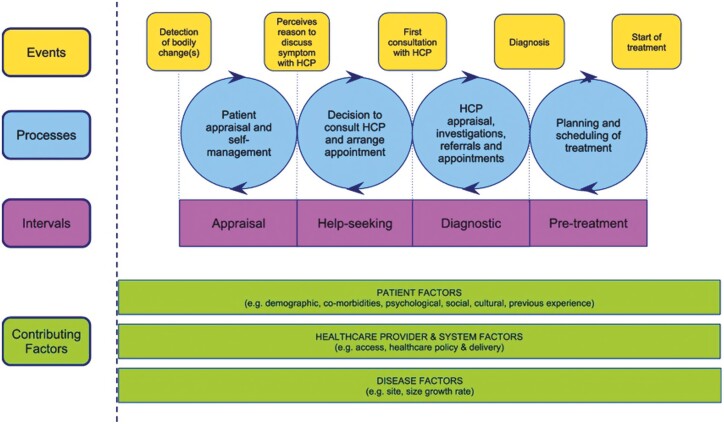
The model of pathways to treatment (MPT).^9^

The MPT^9^ was used as the framework for data analysis, focussing on the first 3 MPT intervals: appraisal, help seeking, and diagnostic (see Fig. 1). Three intervals were calculated, guided by the Aarhus statement^29^ and a previous study.^25^ We combined the appraisal/help seeking interval, defined as the period from patient-reported first symptom recognition (first notice of body changes or symptoms) to date of first GP presentation or ED admission (when a clinician starts investigations or referral). The GP diagnostic interval was calculated as the date of first GP consult/ED admission to date of diagnosis (defined as patient-reported date of confirmation or date of colonoscopy if patient-reported dates were not certain) and the total diagnostic interval (TDI) was taken as the overall time frame, from date of first symptom onset to date of diagnosis. Delay in each interval was defined as >120 days and no delay was classified as <120 days, based on Australian clinical guidelines.^30^

### Data analysis

Descriptive statistics were used to describe the characteristics of the study population. Factors associated with the patient interval, the GP diagnostic interval, and the TDI were tested using chi-square analysis. Logistic regression was used to analyse factors influencing diagnostic intervals, including age, gender, ethnicity, first reported symptom, and route to diagnosis. Tests for significance were 2 tailed with *P* < 0.001 considered statistically significant. Analyses were performed using SPSS version 27 (New York).

## Results

Two hundred and thirty-five patients were recruited in total, 142 from Waikato, 15 from Tairawhiti, and 60 from Lakes DHBs. Eighteen patients were recruited through NZ Bowel Cancer. Patients were excluded if they had been diagnosed through regional bowel screening (*n* = 7), were more than 12 months post diagnosis (*n* = 32), and had a non-CRC diagnosis (*n* = 1). Following these exclusions, 195 patients remained.

Table 1 shows the characteristics of the study population. The majority (74.9%) of patients were aged >60, non-Māori (84.6%) and male (55.9%). A single, first patient-reported symptom prior to diagnosis was experienced by 145 (74.4%) patients, and multiple (i.e. 2–5) first-noticed symptoms were reported by 39 (20.0%) patients. COBH was the most common symptom across the whole cohort, reported by 123 (63.1%) patients, followed by rectal bleeding, reported by 108 (55.4%) patients. However, the most common first-noticed, patient-reported symptom was rectal bleeding (31.8%) followed by COBH (26.7%). Due to small numbers, weight loss, fatigue, and loss of appetite were combined in an “other” symptom category. When asked if they had reported their symptom(s) to a GP or nurse, 36 (19.6%) patients did not report their COBH, and 17 (9.2%) did not report rectal bleeding. The most common diagnostic pathway was through general practice (64.1%), followed by ED admission (15.4%). Forty patients were diagnosed through an incidental or “other” pathway, 19 of which were removed from all further analyses as they had not been diagnosed as a result of a visit with their GP (e.g. they were already in hospital for a non-related reason or were being monitored due to a family history of bowel cancer or polyps). Eleven patients (5.6%) reported zero symptoms, of which 7 pateints were diagnosed either incidentally or through an “other” pathway (i.e. and so were part of those cases excluded), leaving 4 patients who reported zero symptoms but were diagnosed following a routine visit with their GP. The removal of these cases left a sample size of 176 for further analysis.

**Table 1.** Characteristics of patients diagnosed with CRC in the Midland region of NZ (2016–2019) (*N* = 195).

Factors	n	%
Age group		
<60	49	25.1
60+	146	74.9
Ethnicity		
Non-Māori	165	84.6
Māori	29	14.9
Missing	1	0.5
Gender		
Male	109	55.9
Female	86	44.1
Comorbidities		
0	74	37.9
1+	121	62.1
Number of first-reported symptoms		
0	11	5.6
1	145	74.4
2+	39	20.0
First-reported symptom		
COBH^a^	52	26.7
Bleeding	62	31.8
Abdominal/anal pain	32	16.4
Other^b^	38	19.5
Zero reported symptoms	11	5.6
Diagnostic pathway		
GP	125	64.1
Incidental	29	14.9
ED	30	15.4
Other	11	5.6
Did a GP refer for colonoscopy?		
No	72	36.9
Yes	108	55.4
NA/missing/don’t know	15	7.7

COBH = change of bowel habit.

Other = bloating, vomiting, nausea, iron deficiency, anaemia, dizziness, appetite loss.

### Appraisal/help-seeking interval

Table 2 shows the cohort characteristics stratified by appraisal/help-seeking, GP diagnostic, and TDI. Only 35 (19.0%) patients appraised symptoms and engaged in help-seeking > 120 days. Of these, 6 (17.1%) had been experiencing rectal bleeding. Patients who delayed seeking medical help were more likely to be <60 (*P* = 0.362), male (*P* = 0.264), although these factors were not significant according to a chi-square analysis. Significant factors in this phase were a COBH as a first-noticed symptom (*P* < 0.001) and route to diagnosis (*P* < 0.001).

**Table 2.** The characteristics of all patients diagnosed with CRC in the Midland region of NZ (2016–2019), stratified by appraisal/help-seeking, GP diagnostic, and total diagnostic interval (TDI) (*n* = 176).

	Appraisal/help-seeking Interval						GP diagnostic interval						Total diagnostic interval				
Factors	<120 days		>120 days		Unknown		<120 days		>120 days		Unknown		<120 days		>120 days		
	*n* = 129	%	*n* = 35	%	*n* = 8	*p*	*n* = 99	%	*n* = 65	%	*n* = 8	*p*	*n* = 76	%	*n* = 96	%	*p*
Age group																	
<60	32	24.8	12	34.3	3	0.362	26	26.3	18	27.7	3	0.577	17	22.4	30	31.3	0.229
60+	97	75.2	23	65.7	5		73	73.7	47	72.3	5		59	77.6	66	68.8	
Ethnicity																	
Non-Māori	113	87.6	30	85.7	3	0.003	90	90.9	53	81.5	3	0.004	68	89.5	78	81.3	0.232
Māori	16	12.4	4	11.4	5		9	9.1	11	16.9	5		8	10.5	17	17.7	
Missing	0	0.0	1	2.9	0		0	0.0	1	1.5	0		0	0.0	1	1.0	
Gender																	
Male	68	52.7	21	60.0	4	0.264	61	61.6	28	43.1	4	0.031	45	59.2	48	50.0	0.281
Female	61	47.3	14	40.0	4		38	38.4	37	56.9	4		31	40.8	48	50.0	
Comorbidities																	
0	50	38.8	13	37.1	3	0.954	40	40.4	23	35.4	3	0.869	30	39.5	36	37.5	0.875
1+	79	61.2	22	62.9	5		59	59.6	42	64.6	5		46	60.5	60	62.5	
First reported symptom																	
COBH^a^	32	24.8	15	42.9	0	**<0.001**	27	27.3	20	30.8	0	**<0.001**	16	21.1	31	32.3	0.123
Bleeding	50	38.8	6	17.1	5		40	40.4	16	24.6	5		34	44.7	27	28.1	
Abdominal/anal pain	24	18.6	6	17.1	2		17	17.2	13	20.0	2		14	18.4	18	18.8	
Other^b^	23	17.8	8	22.9	1		15	15.2	16	24.6	1		12	15.8	20	20.8	
Zero symptoms	0	0.0	0	0.0	0		0	0.0	0	0.0	0		0	0.0	0	0.0	
Diagnostic pathway																	
GP	94	72.9	26	74.3	5	**<0.001**	72	72.7	48	73.8	5	**<0.001**	53	69.7	72	75.0	0.648
Incidental	9	7.0	2	5.7	2		6	6.1	5	7.7	2		6	7.9	7	7.3	
ED	22	17.1	7	20.0	1		20	20.2	9	13.8	1		16	21.1	14	14.6	
Other	4	3.1	0	0.0	0		1	1.0	3	4.6	0		1	1.3	3	3.1	

Bold values represent statistically significant results.

COBH = change of bowel habit.

Other = bloating, vomiting, nausea, iron deficiency, anaemia, dizziness, appetite loss.

### GP diagnostic interval

For the GP diagnostic interval, 65 (36.9%) patients experienced an interval of >120 days. Patients who experienced longer intervals during this phase were more likely to be Māori (*P* = 0.004) and female (*P* = 0.031). ED admission, or being diagnosed through an incidental or “other” finding were significantly faster routes to diagnosis (*P* < 0.001). Patients who had experienced a COBH (*P* < 0.001) and route to diagnosis were significant factors in this phase (*P* < 0.001).

### Total diagnostic Interval

A TDI with known dates was calculated for 176 patients. Over half (54.5%) of patients experienced a TDI > 120 days. All factors were non-significant according to a chi-square analysis.

The median TDI across the whole cohort was 142 days (IQR 61–365), 30 days (IQR 0–93) for the appraisal/help-seeking interval, and 66 days (IQR 26–236) for the GP diagnostic interval. Patients aged <60 had a higher median TDI (239 days) than those aged 60+ (122 days) (see Table 3). Māori, and female patients had a longer median TDI and GP diagnostic interval (Māori TDI: 231 days; GP diagnostic: 170 days—females TDI: 160 days; GP diagnostic: 120 days) compared to non-Māori and males. ED presentation had the shortest median days across all intervals (TDI: 108 days; appraisal/help seeking: 1 day; GP diagnostic: 48 days), as did rectal bleeding (TDI: 104 days; appraisal/help seeking: 16 days; GP diagnostic: 54 days), with the exception of the appraisal/help seeking phase, where abdominal or anal pain had the shortest median days to diagnosis (8 days).

**Table 3.** Median number of days patients spent in the appraisal/help-seeking, GP diagnostic, and total diagnostic intervals (TDI) (*n* = 176).

	Appraisal/help-seeking interval	GP diagnostic interval	Total diagnostic interval	Totals
Factors	Median (IQR)	Median (IQR)	Median (IQR)	n
Age group				
<60	30 (0–138)	64 (30–345)	239 (61–587)	129
60+	30 (0–92)	67 (24–194)	122 (61–365)	136
Ethnicity				
Non-Māori	30 (0–92)	62 (25–197)	127 (61–362)	149
Māori	22 (0–109)	170 (15–451)	231 (107–681)	26
Missing	—	—	—	1
Gender				
Male	30 (2–108)	53 (15–170)	122 (61–338)	97
Female	30 (0–92)	120 (38–334)	160 (65–635)	79
Comorbidities				
0	30 (1–92)	61 (28–203)	142 (60–355)	70
1+	30 (0–100)	86 (24–256)	146 (61–370)	114
First reported symptom				
COBH^a^	35 (14–181)	90 (30–231)	194 (91–638)	47
Bleeding	16 (0–47)	54 (17–130)	104 (49–304)	61
Abdominal/anal pain	8 (0–94)	93 (7–206)	138 (49–297)	32
Other^b^	61 (7–127)	165 (21–344)	313 (76–653)	32
Zero symptoms^c^	na	na	na	4
Diagnostic pathway				
GP	31 (14–105)	75 (28–260)	151 (64–365)	125
Incidental	0 (0–10)	65 (32–1,096)	130 (53–914)	16
ED	1 (0–117)	48 (3–154)	108 (30–365)	30
Other	4 (0–24)	501 (110–1,088)	519 (117–1,089)	5

COBH = change of bowel habit.

Other = bloating, vomiting, nausea, iron deficiency, anaemia, dizziness, appetite loss.

Time frames for each interval could not be calculated for patients with zero symptoms as they had no period of symptom onset.

After adjusting for all factors, patients reporting rectal bleeding were less likely to experience a long TDI (odds ratio [OR] 0.34, 95% confidence interval [CI]: 0.14–0.78) and appraisal/help-seeking interval (OR, 0.19, 95% CI: 0.06–0.59). Compared with patients aged >60, younger patients were more likely to experience longer appraisal/help-seeking intervals (OR, 3.32, 95% CI: 1.17–9.46) and females (OR, 2.19, 95% CI: 1.08–4.44) and Māori patients (OR, 3.18, 95% CI: 1.04–9.78) were more likely to experience a long GP diagnostic interval.

## Discussion

Around 1,200 New Zealanders a year die from bowel cancer. Survival from colon and rectal cancer in NZ is 64.5% and 69.3%, respectively (2010–2014)^31^ and has not been improving. While the new NZ screening programme is likely to help, the age cut off for the programme is 60–74, and with limited uptake, the impact will be modest. Therefore, addressing delay in diagnosis will still be important. Intervals to CRC diagnosis were investigated according to 3 phases of the MPT (appraisal, help seeking, diagnostic). Over half of the cohort experienced a TDI of more than 120 days. As expected from a largely unscreened population, most patients were diagnosed through general practice. Rectal bleeding and COBH were the most common first-noticed, patient-reported symptoms. Rectal bleeding was associated with a shorter appraisal/help-seeking, GP diagnostic, and TDI. Younger patients experienced longer times across all intervals and Māori and female patients were more likely to experience a longer GP diagnostic interval.

Twenty-five percent of the cohort were aged <60, supporting the growing observation in NZ and internationally that CRC incidence is increasing in younger age groups.^32,33^ Younger patients delayed seeking medical help beyond 120 days, perhaps consistent with public perceptions that CRC more commonly affects older people. Compared with rectal bleeding, patients who first reported a COBH delayed consulting a GP, and almost 20.0% never reported their COBH. This likely reflects difficulty in discriminating bowel changes from more serious conditions, especially if individuals have pre-existing GI issues or consider irregular bowels as “normal.” Similar to other studies,^25,34^ we found that the more overt, “red flag” symptoms of rectal bleeding and abdominal pain were associated with shorter time frames, especially if bleeding was sudden or abdominal pain was related to obstruction resulting in an ED admission. Also consistent with other research,^26^ patients reported not appreciating symptom seriousness, being reassured by an alternative GP diagnosis, and not feeling alarmed about symptoms if they had been previously experienced. Given similar findings from another NZ study^34^ and CRCs historically low profile,^35^ we need to increase CRC education regarding recognition of signs and symptoms of CRC to improve knowledge and reduce opportunities for delay.

Compared with other cancers, CRC is associated with a longer diagnostic interval in general practice,^36^ reflecting the difficulty in diagnosis.^37^ We report a long GP-related interval for 36.9% of patients from first GP consult to diagnosis and a TDI for 54.5%, with a median 142 days from symptom onset to diagnosis. Patients were more likely to experience a longer GP diagnostic interval if they were Māori, female, or reported a COBH as their first-noticed symptom. Māori have a lower incidence of CRC than NZ Europeans,^38^ but experience greater inequity accessing health services,^39^ less choice of GP appointments,^40^ and less access to chemotherapy^41^ and colonoscopy.^42^ Our findings for Māori are consistent with other NZ CRC studies,^34,43^ but, as with those studies, are limited by a small sample size. That said, there is demand for urgent action addressing the inequity of the national bowel screening programme—with the age set at 60 which ignores the higher number of Māori diagnosed with CRC at a younger age, and contributes to their poorer outcomes.^44^ While men are more likely to develop CRC than women,^45^ consistent with studies reporting longer diagnostic intervals for female patients,^8,37^ females in this cohort had a greater GP diagnostic interval than males. Proportionally, females were also less likely to be referred for colonoscopy (57.1% compared with 42.9% males). Some female patients described a “battle,” with GPs misattributing symptoms to B12 deficiency or menopause. Gynaecological issues can confound a CRC diagnosis,^8^ but it is also possible that an unconscious gender bias may be contributing to longer diagnostic intervals for female patients.

COBH was also associated with longer GP-related intervals. With COBH common in the general population and more typically associated with benign conditions, GPs face considerable diagnostic difficulty in discriminating these symptoms from CRC. For example, a systematic review reported the prevalence of IBS across a number of countries as 11.2%.^46^ GPs also face barriers to referring patients for specialist diagnostic tests.^28^ NZs Ministry of Health (MOH) referral guidelines state that a COBH must be present for >6 weeks and accompanied by rectal bleeding in those aged over 50 for a direct access to colonoscopy referral.^47^ Of the total patients who reported a COBH, 78 (63.4%) also had rectal bleeding. Forty-seven (60.3%) of these patients had a TDI >120 days, and 34 (43.6%) had a GP diagnostic interval of >120 days. Some of these patients likely represent missed opportunities for diagnosis. Ongoing review of access criteria is needed to ensure inequities are not worsened; the unintended consequences of generic criteria will often worsen access and outcomes in priority populations (i.e. indigenous people). Likewise, 16 (24.2%) people presented to GPs with rectal bleeding but waited >120 days until diagnosis. Some of these patients were misdiagnosed with haemorrhoids—sometimes without a digital rectal exam (DRE). A failure to conduct DREs was a major source of complaint in the HDC report (2004–2013)^7^ and has been frequently cited as a continuing problem in CRC research.^15,37^ Calls for increased use of DRE in NZ are not new,^48^ yet a 2019 NZ study reported no DRE in 42.0% of cases,^49^ suggesting failure to perform DREs remains an ongoing issue. Another option to reduce missed diagnoses is the faecal immunochemical test (FIT), a widely used, non-invasive test that can function as a diagnostic step to colonoscopy.^50^ NICE guidelines^51^ recommend FIT to discriminate those with non-specific abdominal pain and/or COBH, but access to FIT is not available to GPs in the NZ health system outside bowel screening. Consequently, NZ GPs cannot use FIT for symptomatic triage of CRC. With red flag symptoms associated with less delay to diagnosis, it is even more important that GPs—who play an especially important role in the early diagnosis of cancer—have appropriate access to diagnostic tools such as FIT.

Data were collected from a large region in NZ, however, sample size is a limitation. A weakness of questionnaires is that participants may not fully understand or answer questions appropriately. To minimize this risk, data collection was researcher-assisted. However, data were still patient-reported, and while interviews were conducted as close to diagnosis date as possible (within 12 months of diagnosis), recall bias may have been an issue. Patient and provider reports of diagnostic time-points can also differ.^35^ The questionnaire did not ask for reporting on conditions such as ulcerative colitis, diverticulitis, or Crohn’s disease, so we could not provide data on numbers with these conditions. Finally, with a focus on general practice, we did not record the number of patients who may have experienced longer intervals waiting for secondary care appointments (e.g. colonoscopy). In addition, system factors including GP access to diagnostic tests and their impact on TDI were unable to be assessed.

## Conclusions

Many NZ patients newly diagnosed with CRC experience long diagnostic intervals, attributed to a combination of patient and health care provider factors. Young patients, Māori, females, and patients experiencing a COBH may be at risk for greater chance of delay. With the diagnostic difficulty of CRC, we need to increase the public profile of CRC and symptom awareness for both patients and GPs. There needs to be concentrated efforts to ensure equity for Māori.

## Supplementary Material

cmab155_suppl_Supplementary_Material

## Data Availability

The data underlying this article are available in the article and in its Supplementary Material.
