# Supplementary material for: Patient-reported diagnostic intervals to colorectal cancer diagnosis in the Midland region of New Zealand: a prospective cohort study
Source: Fam Pract. 2021 Dec 6;39(4):639–47. doi: 10.1093/fampra/cmab155 (PMC9295611; doi:10.1093/fampra/cmab155)
Supplement: cmab155_suppl_Supplementary_Material [file cmab155_suppl_supplementary_material.docx]

Title and abstract

1. Indicate the study’s design with a commonly used term in the title or the abstract

*We have identified the study design as a prospective cohort study in the* ***Title***

1. Provide in the abstract an informative and balanced summary of what was done and what was found

*We have provided an informative and balanced summary in the* ***Abstract***

Introduction

Background/rationale

Explain the scientific background and rationale for the investigation being reported

*This has been explained in the* ***Background*** *section*

Objectives

State specific objectives, including any prespecified hypotheses

*The objective of the study has been stated in the last sentence of the* ***Background*** *section*

Methods

Study design

Present key elements of study design early in the paper

*The study design has been fully described in the* ***Methods*** *section*

Setting

Describe the setting, locations, and relevant dates, including periods of recruitment, exposure, follow-up, and data collection

*This information can be found in the* ***Patient recruitment*** *section of the* ***Methods***

Participants

1. Give the eligibility criteria, and the sources and methods of selection of participants. Describe methods of follow-up

*Patient recruitment has been described in the* ***Patient recruitment*** *section. Follow-up is not relevant to this study*

1. For matched studies, give matching criteria and number of exposed and unexposed

*Not relevant to this study*

Variables

Clearly define all outcomes, exposures, predictors, potential confounders, and effect modifiers. Give diagnostic criteria, if applicable

*The questionnaire used has been described. Questionnaires were researcher-assisted to avoid subjective responding. This has been mentioned in the* ***Discussion*** *section. Diagnostic criteria are not applicable*

Data sources/ measurement

For each variable of interest, give sources of data and details of methods of assessment (measurement). Describe comparability of assessment methods if there is more than one group

*Data collection and assessment has been described in the* ***Methods*** *section.*

Bias

Describe any efforts to address potential sources of bias

*Bias is not relevant to this study per se. However, questionnaires were researcher assisted to reduce respondent bias. This is mentioned in the* ***Discussion*** *section*

Study size

Explain how the study size was arrived at

*Study size has been described in the* ***Patient recruitment*** *section of the* ***Methods****, and the first paragraph of the* ***Results*** *section*

Quantitative variables

Explain how quantitative variables were handled in the analyses. If applicable, describe which groupings were chosen and why

*Calculation of diagnostic dates have been described in the* ***Data collection*** *section of the* ***Methods****, and calculation of delay intervals and data analysis have also been described in the* ***Methods section***

Statistical methods

1. Describe all statistical methods, including those used to control for confounding

*Statistical methods have been described in the* ***Data analysis*** *section of the* ***Methods***

(b) Describe any methods used to examine subgroups and interactions

*We have mentioned using chi-square analysis and logistic regression to analyse factors influencing diagnostic interval. This can be found in the* ***Data analysis*** *section of the* ***Methods***

(c) Explain how missing data were addressed

*Not applicable*

(d) If applicable, explain how loss to follow-up was addressed

*Not applicable*

(e) Describe any sensitivity analyses

*Not applicable to this study*

Results

Participants

1. Report numbers of individuals at each stage of study—eg numbers potentially eligible, examined for eligibility, confirmed eligible, included in the study, completing follow-up, and analysed

*This information has been described in the* ***Patient recruitment*** *section of the* ***Methods****. Numbers of individuals in the analysis has been reported in the* ***Results*** *section*

(b) Give reasons for non-participation at each stage

*Eligibility has been described in the* ***Patient recruitment*** *section of the* ***Methods****. Exclusion criteria have been described in the first paragraph of the* ***Results*** *section*

(c) Consider use of a flow diagram

*Figure 1, in the* ***Methods*** *section, describes the Model of Pathways to Treatment, and is a type of flow diagram.*

Descriptive data

(a) Give characteristics of study participants (eg demographic, clinical, social) and information on exposures and potential confounders

*Patient characteristics have been described in Tables 1 and 2 in the* ***Results*** *section*

(b) Indicate number of participants with missing data for each variable of interest

*Number of participants has been stated in the* ***Results*** *section*

(c) Summarise follow-up time (eg, average and total amount)

*Follow up time is not relevant to this study*

Outcome data

Report numbers of outcome events or summary measures over time

*All outcomes have been reported in* ***Tables 2-3*** *and in the* ***Results*** *section*

Main results

(a) Give unadjusted estimates and, if applicable, confounder-adjusted estimates and their precision (eg, 95% confidence interval). Make clear which confounders were adjusted for and why they were included

*Regression after adjustment for confounding factors has been stated, with 95% confidence intervals in the* ***Results*** *section. Due to a small sample size, we have only reported the significant adjusted results here*

(b) Report category boundaries when continuous variables were categorized

*Not applicable*

1. If relevant, consider translating estimates of relative risk into absolute risk for a

meaningful time period

*Not applicable*

Other analyses

Report other analyses done—eg analyses of subgroups and interactions, and sensitivity analyses

*All analyses have been reported in the* ***Results*** *section, including descriptive statistics, chi-square analyses and regression*

Discussion

Key results

Summarise key results with reference to study objectives

*Key results have been summarised at the start of the* ***Discussion*** *section*

Limitations

Discuss limitations of the study, taking into account sources of potential bias or imprecision. Discuss both direction and magnitude of any potential bias

*Strengths and limitations have been identified in the* ***Discussion*** *section. Bias is not relevant to the current study*

Interpretation

Give a cautious overall interpretation of results considering objectives, limitations, multiplicity of analyses, results from similar studies, and other relevant evidence

*These points have been covered in the* ***Discussion*** *section*

Generalisability

Discuss the generalisability (external validity) of the study results

*Generalisability has been alluded to in the* ***Discussion*** *section* *and the small sample size has been mentioned as a limitation, even though data was collected from a fairly large geographical region.*

Other information

Funding

Give the source of funding and the role of the funders for the present study and, if applicable, for the original study on which the present article is based

*The funding source has been stated in the Funding statement in the* ***Declarations*** *section*
